# Predictors of COVID-19 Mortality in Critically Ill ICU Patients: A Multicenter Retrospective Observational Study

**DOI:** 10.7759/cureus.20952

**Published:** 2022-01-05

**Authors:** Chukwuemeka Umeh, Laura Tuscher, Sobiga Ranchithan, Kimberly Watanabe, Rahul Gupta

**Affiliations:** 1 Internal Medicine, Hemet Global Medical Center, Hemet, USA

**Keywords:** tachycardia, crp, mortality, icu, sars-cov-2, covid-19

## Abstract

Introduction

Coronavirus disease 2019 (COVID-19) is a multisystemic disease caused by severe acute respiratory syndrome coronavirus 2 (SARS-CoV-2) and can lead to a broad spectrum of disease severity, from asymptomatic to severe respiratory disease. In addition, the mortality rate is exceedingly high among COVID-19 patients admitted to the ICU. The purpose of this study is to examine the differences between survivors and non-survivors of critically ill COVID-19 patients admitted to the ICU.

Method

This multicenter retrospective observational study was conducted at two hospitals in Southern California, USA. First, we compared the characteristics of the ICU patients that died and those that survived using the chi-square test for categorical variables and t-test for the continuous variables, with a p-value of 0.05 considered significant. Finally, we did a backward selection Cox multivariate regression analysis using mortality as a dependent variable.

Result

There were 1,116 patients admitted with COVID-19 during our study period. Of this number, 238 (21.3%) were admitted to the ICU. Among patients admitted to the ICU, 195 (81.9%) died and 43 (18.1%) survived. In the multivariate Cox regression analysis, C-reactive protein (CRP) (HR 1.03, 95% CI 1.003-1.059), tachycardia (HR 3.51, 95% CI 1.83-6.72), and age (HR 1.02, 95% CI 1.01-1.04) were independently associated with mortality. Patients’ BMI and comorbidities such as hypertension, diabetes, chronic obstructive pulmonary disease, and chronic kidney disease did not predict mortality.

Conclusion

Age, elevated CRP, and tachycardia were independent risk factors for mortality in COVID-19 patients admitted to the ICU. It appears that several factors that predict severe diseases in COVID-19 patients, such as BMI and comorbidities, become less important once patients are admitted to the ICU.

## Introduction

Coronavirus disease 2019 (COVID-19) refers to the disease caused by severe acute respiratory syndrome coronavirus 2 (SARS-CoV-2). The novel virus first emerged in December 2019 and quickly spread worldwide, resulting in the COVID-19 pandemic. The virus is transmitted via respiratory droplets and primarily uses angiotensin-converting enzyme 2 (ACE2) receptors throughout the body to produce a multisystemic infection [1, 2]. Symptoms range from mild to severe, with fatigue and expectoration being two of the most important prognostic factors of severe disease [3]. In addition, factors such as age and the presence of comorbidities also affect the severity and outcome of COVID-19 [4]. The severity of COVID-19 has also been linked to the development of a cytokine storm, with an overwhelming and dysfunctional release of cytokines. In addition, multiple cytokines are produced at higher levels in patients with severe disease than those with mild or moderate illness [5].

Patients with severe symptoms may require ICU admission for critical care and mechanical ventilation support [6]. Critical conditions may include severe interstitial pneumonia and acute respiratory distress syndrome, thrombotic conditions such as pulmonary embolism, and severe disease may result in direct or indirect cardiac complications as well as a multitude of neurological complications [7]. In addition, several studies have reported differences in mortality in patients admitted to the ICU in different centers [8]. It is possible that the differences in mortality in ICU patients and the factors that affect mortality are influenced by patients’ demographics, ICU availability, admission policy, among others. Thus, it is likely that the factors that affect ICU outcomes will vary by location. The purpose of this study is to examine the differences between survivors and non-survivors of ICU patients with COVID-19 in two hospitals in California. Understanding the ICU outcome predictors will help clinicians risk-stratify the patients admitted to the ICU and in clinical decision-making.

## Materials and methods

This multicenter retrospective observational study was conducted at two hospitals in Southern California, USA. The study included all 238 consecutive COVID-19 patients admitted to the ICU between March 2020 and March 2021. All patients were confirmed to have COVID-19 infection through a positive PCR nasopharyngeal swab. Each patient was seen by a COVID-19 team consisting of a pulmonary and critical care specialist and hospitalists in consultation with an infectious disease specialist and pharmacist.

We extracted relevant de-identified patient data from the electronic medical record using a structured query language (SQL) program, which included: age, gender, BMI on admission, race, comorbidities, laboratory results on hospital admission, date of admission, date of discharge, medications they received while on admission, such as beta-blockers, statin, steroid, angiotensin-converting enzyme inhibitors (ACEi), etc., heart rate, and disposition at discharge. We divided the patients into two groups: those that died and those that survived. Furthermore, we defined tachycardia as a sustained heart rate >100 beats per minute on two separate occasions, a minimum of 4 hours apart during the hospitalization. Our primary outcome was predictors of mortality for ICU patients. The secondary outcomes included length of hospital stay and mechanical ventilation by those that died and those that did not.

We compared the characteristics of the two groups using the chi-square test for categorical variables and t-test for the continuous variables, with a p-value of 0.05 considered significant. Finally, we did a backward selection Cox multivariate regression analysis using mortality as a dependent variable. We initially included biologically plausible or statistically significant variables from the bivariate analysis, such as patients’ age, sex, C-reactive protein (CRP), lactate dehydrogenase (LDH), d-dimer, tachycardia, and mechanical ventilation, as independent variables in the multivariate model. The effect was expressed in terms of HR, and hypothesis testing was done using a two-sided test, and an alpha value of 0.05 indicated statistical significance. Statistical analysis was done using IBM SPSS version 27 (IBM, New York, USA). The study was approved by the WIRB-Copernicus Group (WCG) institutional review board, and the study IRB approval number is 13410516.

## Results

There were 1,116 patients admitted with COVID-19 during our study period. Of this number, 238 (21.3%) were admitted to the ICU. Among patients admitted to the ICU, 195 (81.9%) died and 43 (18.1%) survived. The patients that died were significantly older than those that survived (p=0.012). However, there was no difference in the BMI of the patients that died and those that survived (p=0.95). CRP (p=0.015) and d-dimer (p=0.018) were significantly higher in those that died than those that survived. Conversely, lactate dehydrogenase (LDH) (p=0.056), ferritin (p=0.42), troponin (p=0.26), sex (p=0.50), and race (p=0.76) were not different in those that died and those that survived. The incidence of tachycardia (p<0.001) and acute kidney injury (p<0.001) were higher in patients that died than those that survived (Table 1).

**Table 1:** Characteristics of COVID-19 patients admitted to the ICU

Variable	All ICU patients [mean (standard deviation)] (n =238)	Survived [mean (standard deviation)] (n = 43)	Died [mean (standard deviation)] (n = 195)	P-value
Age (years)	65.88 (14.14)	59.74 (17.83)	67.24 (12.86)	0.012
BMI (kg/m^2^)	31.66 (9.54)	31.74 (9.43)	31.64 (9.58)	0.947
C-reactive protein (mg/dl)	11.43 (6.44)	8.85 (6.70)	11.89 (6.30)	0.015
Lactate dehydrogenase (IU/l)	604.02 (665.97)	404.53 (179.44)	643.69 (718.63)	0.056
D-dimer (ng/ml)	1870.97 (1626.46)	1315.36 (1505.16)	1990.69 (1630.63)	0.018
Ferritin (ng/ml)	1235.53 (3386.42)	556.65 (483.06)	1306.23 (3548.88)	0.416
Troponin (ng/ml)	0.43 (1.82)	0.14 (0.32)	0.50 (1.99)	0.259
Sex				
Female	89 (37.4%)	18 (41.9%)	71 (36.4%)	0.504
Male	149 (62.6%)	25 (58.1%)	124 (63.6%)	
Race				
White	186 (78.2%)	33 (76.7%)	153 (78.5%)	0.757
Black	15 (6.3%)	2 (4.7%)	13 (6.7%)	
Others	37 (15.5%)	8 (18.6%)	29 (14.9%)	
Bradycardia (HR <60)	120 (50.4%)	21 (48.8%)	99 (50.8%)	0.819
Severe bradycardia (HR <50)	44 (18.5%)	9 (20.9%)	35 (17.9%)	0.648
Tachycardia (HR >100)	207 (87.0%)	30 (69.8%)	177 (90.8%)	<0.001
Coronary artery disease	64 (26.9%)	11 (25.6%)	53 (27.2%)	0.831
Chronic obstructive pulmonary disease	43 (18.1%)	9 (20.9%)	34 (17.4%)	0.590
Heart failure	56 (23.5%)	11 (25.6%)	45 (23.1%)	0.726
Chronic kidney disease	65 (27.3%)	10 (23.3%)	55 (28.2%)	0.510
Acute kidney injury	113 (47.5%)	10 (23.3%)	103 (52.8%)	<0.001
Hypertension	160 (67.2%)	27 (62.8%)	133 (68.2%)	0.494
Diabetes mellitus	130 (54.6%)	27 (62.8%)	103 (52.8%)	0.235

Patients on mechanical ventilators were more likely to die (p<0.001). Of the 238 ICU patients in our study, 178 (75%) required mechanical ventilation, which amounted to 82% of the dead patients and 42% of the patients that survived. However, some patients who died chose not to be intubated or resuscitated and were not placed on the ventilator. In patients requiring mechanical ventilation, the mortality rate was close to 90%. However, there was no difference in the hospital length of stay for patients that survived and those that died (p=0.84) (Table 2).

**Table 2:** Outcomes of choosing ventilation and the length of stay in COVID-19 patients admitted to ICU

Variable	All ICU patients (n =238)	Survived (n = 43)	Died (n = 195)	P-value
Mechanical ventilation	178 (74.8%)	18 (41.9%)	160 (82.1%)	<0.001
Length of stay (days) (mean (standard deviation))	14.65 (10.74)	14.28 (13.62)	14.73 (10.04)	0.839

In the multivariate Cox regression analysis, including covariates of age, sex, CRP, LDH, d-dimer, tachycardia, ACEi or angiotensin receptor blocker (ARB) use, beta-blocker use, and mechanical ventilation, CRP (HR 1.03, 95% CI 1.003-1.059), tachycardia (HR 3.51, 95% CI 1.83-6.72), and age (HR 1.02, 95% CI 1.01-1.04) were independently associated with mortality (Table 3).

**Table 3:** Cox multivariate regression analysis of predictors of mortality in COVID-19 patients admitted to ICU ACEi: angiotensin-converting enzyme; ARB: angiotensin receptor blocker; CI: confidence interval; CRP: C-reactive protein

	B	SE	Wald	Sig.	HR	95.0% CI for HR	
						Lower	Upper
CRP	0.030	0.014	4.606	0.032	1.030	1.003	1.059
Use of ACEi or ARB	0.373	0.222	2.833	0.092	1.453	0.940	2.244
Tachycardia	1.255	0.332	14.295	0.000	3.506	1.830	6.719
Age	0.023	0.007	10.036	0.002	1.024	1.009	1.039

## Discussion

Our study looked at the difference between survivors and non-survivors of COVID-19 in patients admitted to ICU in two hospitals in Southern California. Of the 238 patients admitted to the ICU during the study period, 82% died. The ICU mortality rate is higher than the 60% mortality rate reported in two earlier studies [9,10], 42% reported in a meta-analysis of 24 studies [8], and 25% reported in a meta-analysis of 15 studies [11]. However, one of the limitations of the meta-analysis was that many patients in the primary studies included in the meta-analysis were still in the ICU at the time of publication of the study [11]. This study’s high ICU mortality rate may be due to ICU admission being reserved for critically ill patients. Due to pandemic pressures on limited ICU services, noninvasive ventilation and high-flow nasal cannula were widely used outside ICUs, and admission to ICU was reserved for very severe cases [8]. In our study, 75% of those admitted to ICU were placed on a mechanical ventilator, and 90% of those placed on the ventilator died. Thus, patients admitted to the ICU were more likely to undergo mechanical ventilation and more likely to die. This finding is similar to a large study in New York, USA, that showed mortality rates for those who received mechanical ventilation to be 76% in the 18-to-65 age group and 97% in those older than 65 years [12]. Additionally, the socioeconomic and health condition of the patient population that use the hospitals may also be contributory. For example, mortality in COVID-19 has been associated with comorbidities and older age, and hospitals that serve older and sicker patients will have increased mortality.

While many inflammatory markers, including CRP, LDH, and d-dimer, were associated with mortality in the bivariate analysis, only CRP was independently associated with mortality in the multivariate analysis. CRP is an acute-phase protein produced by the liver and has an important role in the inflammatory process provided by growing evidence. CRP assists with the host response to infection, including phagocytosis, complement pathway, nitric oxide (NO) release, and cytokines production, especially interleukin-6 and tumor necrosis factor-α [13]. Additionally, cytokine storms have been suggested to play essential roles in COVID-19 disease severity, and elevated CRP may indicate a more severe form of COVID-19 [14]. While monitoring, CRP may help determine an increased risk of mortality, however, in clinical practice, an increase in any inflammatory markers should raise concern for increased mortality risk, especially in facilities that do not have access to CRP.

Tachycardia was associated with mortality in our patients. Patients who had a heart rate of 100 beats per minute were 3.5 times more likely to die than those who did not. Tachycardia response is expected with infection, inflammation, or fever, and sinus tachycardia has been reported as the most common cardiac abnormality in COVID-19 [15]. Previous studies have shown that sinus tachycardia and ventricular arrhythmia were independent risk factors for increased mortality in COVID-19 patients [14]. In our study, 91% of those who died had tachycardia compared to 70% of those that survived. Our numbers are much higher than the sinus tachycardia rates of 24% and 7% reported by Li et al. in COVID-19 ICU patients that died and survived, respectively [14]. However, our patients had 24-hour telemetry monitoring, unlike most patients in Li et al.’s study, which might explain our higher tachycardia rate. Additionally, ICU hyperthermia, which causes tachycardia, is predictive of mortality in ventilated COVID-19 patients. Increased mortality persists irrespective of targeted temperature management to achieve normothermia in these patients [9].

Finally, while older age, increase in inflammatory markers, and tachycardia are predictors of mortality in ICU patients, it appears that several factors that predict severe disease in COVID-19 patients become less important once patients are admitted to the ICU. For example, our study showed that BMI and comorbidities such as diabetes, hypertension, and chronic kidney disease were not predictors of mortality in ICU patients. The reason for this is unclear, but one possible explanation is that the extent of the cytokine storm and the degree of the damages done to the organs overshadow other factors in critically ill patients.

There are limitations to this study. First, there are a limited number of patients in the study, which might affect the power of the study to detect an effect where one exists. Second, this was a retrospective observational study, and there might have been unmeasured confounders that might have affected our study’s outcome.

## Conclusions

This retrospective observational study provides characteristics and outcomes of sequentially hospitalized ICU patients with confirmed COVID-19 in two hospitals in Southern California. Age, elevated CRP, and tachycardia were independent risk factors for mortality in COVID-19 patients admitted to the ICU. It appears that several factors that predict severe diseases in COVID-19 patients, such as BMI and comorbidities, become less important once patients are admitted to the ICU.
